# Limonin, a Component of Dictamni Radicis Cortex, Inhibits Eugenol-Induced Calcium and cAMP Levels and PKA/CREB Signaling Pathway in Non-Neuronal 3T3-L1 Cells

**DOI:** 10.3390/molecules201219840

**Published:** 2015-12-10

**Authors:** Yeo Cho Yoon, Sung-Hee Kim, Min Jung Kim, Hye Jeong Yang, Mee-Ra Rhyu, Jae-Ho Park

**Affiliations:** 1 Division of Functional Food Research, Korea Food Research Institute, 1201-62 Anyangpangyo-ro, Bundang-gu, Seongnam-si, Gyeonggi-do 463-746, Korea; Yoon.Yeo-cho@kfri.re.kr (Y.C.Y.); Kim.Sung-hee@kfri.re.kr (S.-H.K.); kmj@kfri.re.kr (M.J.K.); yhj@kfri.re.kr (H.J.Y.); mrrhyu@kfri.re.kr (M.-R.R.); 2 Food Biotechnology, University of Science & Technology, 217 Gajeong-ro, Yuseong-gu, Daejeon 305-350, Korea

**Keywords:** limonin, furanolactone, cAMP, olfactory, calcium, dictamni radices cortex

## Abstract

Limonin, one of the major components in dictamni radicis cortex (DRC), has been shown to play various biological roles in cancer, inflammation, and obesity in many different cell types and tissues. Recently, the odorant-induced signal transduction pathway (OST) has gained attention not only because of its function in the perception of smell but also because of its numerous physiological functions in non-neuronal cells. However, little is known about the effects of limonin and DRC on the OST pathway in non-neuronal cells. We investigated odorant-stimulated increases in Ca^2+^ and cAMP, major second messengers in the OST pathway, in non-neuronal 3T3-L1 cells pretreated with limonin and ethanol extracts of DRC. Limonin and the extracts significantly decreased eugenol-induced Ca^2+^ and cAMP levels and upregulated phosphorylation of CREB and PKA. Our results demonstrated that limonin and DRC extract inhibit the OST pathway in non-neuronal cells by modulating Ca^2+^ and cAMP levels and phosphorylation of CREB.

## 1. Introduction

**Figure 1:**
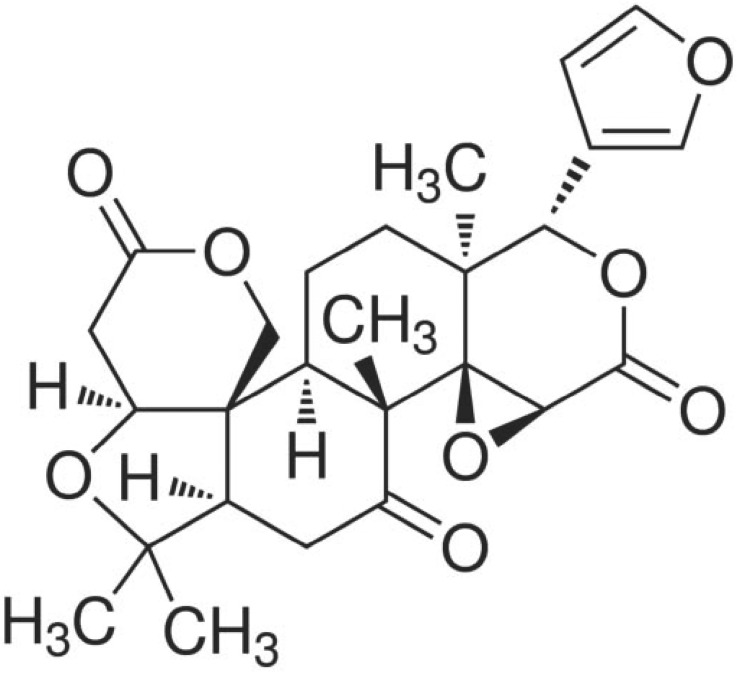
Structure of limonin used in this study.

Dictamni radicis cortex (DRC), a root of *Dictamnus dasycarpus* Turcz, is native to East Asia and has been used as a traditional Korean medicine to treat various diseases, such as allergic dermatitis, jaundice, hepatitis, and rheumatism [1,2]. Using molecular analysis, Sun *et al.* demonstrated the anti-platelet aggregation activity of DRC [3]. Limonin (Figure 1) was first isolated from citrus [4] and is a known component of DRC [5], and many studies have demonstrated its efficacy in the treatment of cancer, inflammation, and obesity [6,7,8]. Limonin affects the expression of lipid metabolism-related genes in 3T3-L1 cells, an *in vitro* model system for the study of adipogenesis [9]. In addition, 3T3-L1 cells are widely used to examine many biological phenomena, such as glucose uptake, apoptosis, and cell cycle [10,11,12]. Yoon *et al.* first demonstrated the utility of 3T3-L1 cells as a model system to study odorant-induced signal transduction (OST) pathway in non-neuronal cells by showing that an olfactory receptor involved in the OST pathway is expressed in 3T3-L1 cells and the OST pathway can be regulated by an exogenous molecule [13].

The olfactory system is necessary for survival, since it is intricately involved in mating, avoidance of predators, fear, and food selection. Olfactory receptors are mainly localized to the cilia on the olfactory sensory neurons (OSN) [14]. The binding of odorants to their receptors is the first step for the perception of smell. The activated receptors increase intracellular cAMP and Ca^2+^ levels, which lead to depolarization of the membrane potential. This signal is transmitted to the brain and is perceived as smell [15,16]. There is emerging evidence demonstrating that ORs ectopically expressed in non-neuronal tissues [17] may have distinct physiological functions [18,19,20,21,22,23]. Importantly, the crucial signaling cascade of cAMP and Ca^2+^ is conserved across neuronal and non-neuronal tissues [19,20,21,22].

In this study, we investigated whether DRC affects the OST pathway in non-neuronal 3T3-L1 cells and determined the amount of limonin in DRC. The effects of limonin on the OST pathway were also examined.

## 2. Results and Discussion

### 2.1. DRC Decreased Ca^2+^ and cAMP Levels in 3T3-L1 Cells

**Figure 2:**
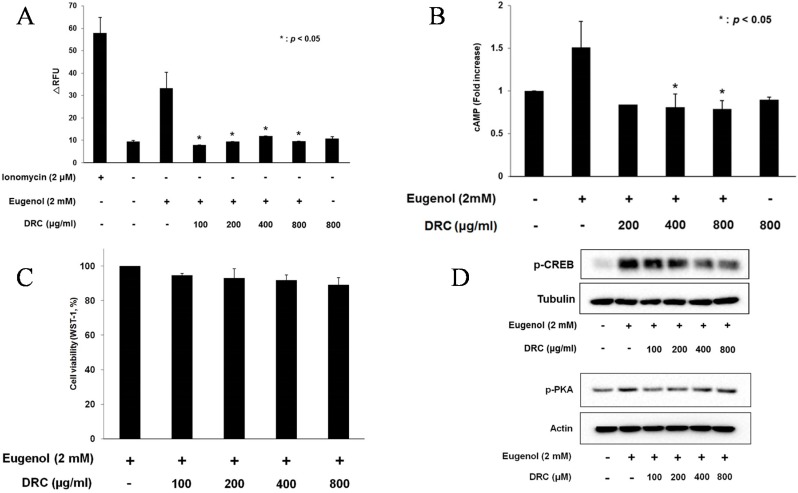
(**A**) The ethanol extracts of dictamni radicis cortex (DRC) decreased Ca^2+^ levels in eugenol-induced 3T3-L1 cells. Ionomycin (2 μM) and DMSO (0.2%) were used as positive and negative controls, respectively. Cells were treated with DRC extracts for 30 min, and Ca^2+^ levels were measured after stimulation with eugenol (2 mM). The data are shown as means ± SD (*n* = 3). *: *p* < 0.05, ΔRFU: change in relative fluorescence unit; (**B**) Changes in cAMP level after pretreatment with the ethanol extracts of dictamni radicis cortex (DRC) for 30 min. The high concentration of DMSO was 0.2% in this experiment. The data are shown as means ± SD (*n* = 3). *: *p* < 0.05; (**C**) Cell viability. 3T3-L1 cells were treated with dictamni radicis cortex (DRC) extracts for 30 min. Cell proliferation was evaluated by WST-8 assay. The data are shown as means ± SD (*n* = 3); (**D**) Phosphorylated protein kinase A (PKA) and cAMP response element binding protein (CREB) protein levels by Western blot in the 3T3-L1 cells after pretreatment with the ethanol extracts of dictamni radicis cortex (DRC) for 30 min. A total of 35 μg of protein was separated by sodium dodecyl sulfate polyacrylamide gel electrophoresis (SDS-PAGE).

Previously, we reported that stimulation with eugenol increased Ca^2+^ and cAMP levels in non-neuronal 3T3-L1 cells [13]. Here, we investigated the effect of ethanol extracts of DRC on the eugenol-induced signaling pathway. In Figure 2A, Ca^2+^ levels dramatically decreased in response to 100–800 μg/mL of DRC extracts while DRC alone without eugenol did not affect calcium level. Since cAMP levels are known to modulate Ca^2+^ levels in the OST pathway [15], we examined the effect of DRC extract on cAMP levels in 3T3-L1 cells. As shown in Figure 2B, eugenol-induced cAMP levels decreased after treatment with 200–800 μg/mL of DRC extracts. In addition, DRC alone did not affect cAMP level. Together with calcium without eugenol, it demonstrated that DRC specifically inhibits eugenol-induced calcium and cAMP increases. Although 100 μg/mL of extracts did not significantly decrease cAMP level (data not shown), it decreased the calcium level. This variation may be owing to differential conditions of the assay systems, including stimulation time, sensitivity of the systems, and temperature at which cAMP and calcium levels were measured. Importantly, 200–800 μg/mL extracts of DRC reduced both measures, Ca^2+^ and cAMP, of OST pathway activation, suggesting that the DRC extracts inhibited the OST pathway in eugenol-induced 3T3-L1 cells. To determine whether these inhibitory effects were due to the toxicity of the extracts, we examined cell viability with extract treatment. As shown in Figure 2C, pretreatment with the extracts (100–800 μg/mL) for 30 min did not affect the proliferation of 3T3-L1 cells, demonstrating that inhibition of Ca^2+^ and cAMP levels by these extracts was not caused by cell toxicity.

### 2.2. DRC Decreased the Phosphorylation of CREB in 3T3-L1 Cells

To investigate the effects of DRC on the OST pathway, phosphorylation of PKA and CREB, known downstream targets of cAMP [23], was carefully examined. As shown in Figure 2D, treatment with DRC extracts (200–800 μg/mL) decreased the phosphorylation of CREB, whereas the phosphorylation of PKA decreased at lower concentration (100–200 μg/mL) and increased at higher concentrations (400 and 800 μg/mL). Although it remains unclear, it is possible that additional components of the ethanol extracts including choline, its derivatives, sitosterol, and campesterol of DRC may differentially affect phosphorylation of these target proteins, which is supported by a report demonstrating that a choline’s derivative can stimulate PKA [24].

### 2.3. Determination of Limonin in the Ethanol Extract of DRC Using UPLC-MS/MS

**Figure 3:**
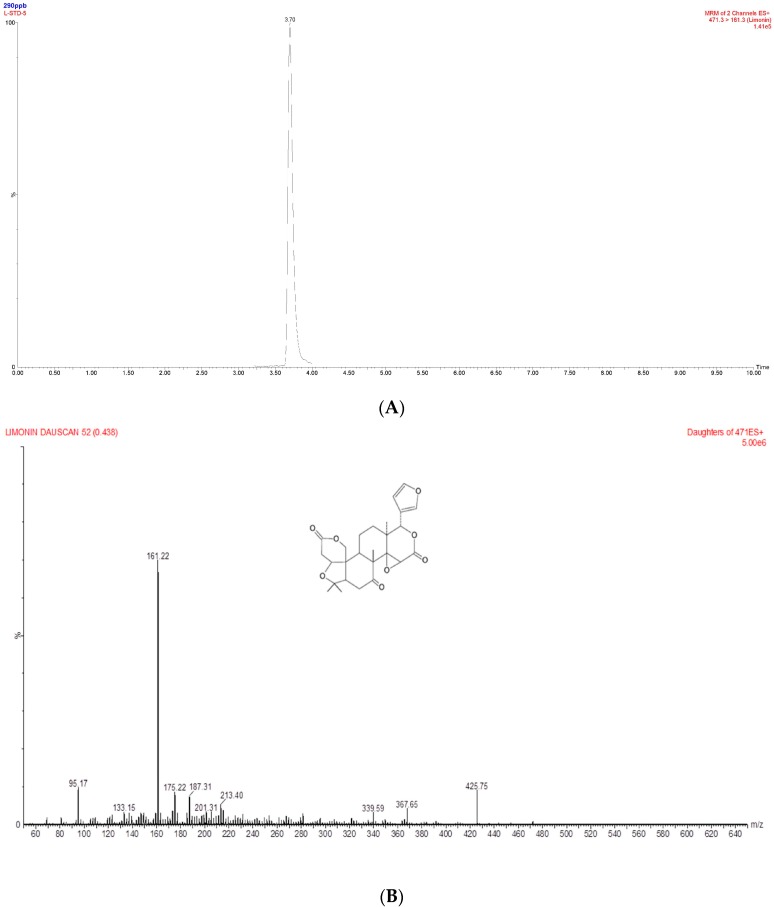
(**A**) Representative UPLC-MS/MS chromatograms of limonin; (**B**) Full-scan product ion spectra of [M + H] + ions and fragmentation pathways for limonin.

Next, we measured the amount of limonin in the DRC extracts using UPLC-MS/MS. As shown in Figure 3A, the retention time of limonin was 3.7 min, and its molecular mass was 470.52 Da. The mass peak was characterized using a mass spectrometer. The parent-to-daughter ion transitions were monitored at *m*/*z* 471.3 → 161.3 (Figure 3B). To determine the concentration of limonin in the extracts, the external calibration curve was first generated with the external limonin. The fitted curve with linear regression was Y = 37.812X + 870.5 with an R^2^ value of 0.981. The limonin content was calculated with the peak area and the calibration curve. The concentration of limonin was 0.216 ± 0.0007 mg/100 g of the ethanol extracts.

### 2.4. Limonin Suppressed Eugenol-Induced Ca*^2+^* and cAMP: Evels in 3T3-L1 Cells

**Figure 4:**
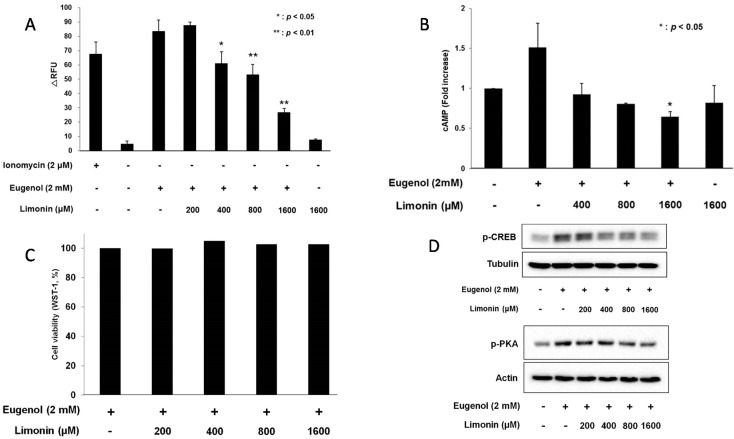
(**A**) Changes of Ca^2+^ levels with limonin pretreatment in eugenol-induced 3T3-L1 cells. Ionomycin (2 μM) and DMSO (0.2%) were used as positive and negative controls, respectively. The data are shown as means ± SD (*n* = 3). *: *p*< 0.05, **: *p*< 0.01, ΔRFU: change in relative fluorescence unit; (**B**) Pretreatment with limonin for 30 min changes cAMP levels in eugenol-induced 3T3-L1 cells. The final concentration of DMSO was 0.2%. The data represent means ± SD (*n* = 3). *: *p* < 0.05; (**C**) Cell viability. 3T3-L1 cells were treated with limonin for 30 min. Cell proliferation was evaluated by WST-8 assay. The data are shown as means ± SD (*n* = 3); (**D**) Phosphorylated protein kinase A (PKA) and cAMP response element binding protein (CREB) levels in 3T3-L1 cells after pretreatment with limonin for 30 min. A total of 35 μg of protein was separated by SDS-PAGE. In experiments for Western blot analysis, the final concentration of DMSO was 0.2%.

In Section 3.3, we showed that limonin is one of the components of DRC extracts; therefore, we investigated the effects of limonin on the OST pathway in 3T3-L1 cells. After pretreatment with limonin, calcium levels were measured under the same conditions as previously described for DRC extracts. As shown in Figure 4A, eugenol-induced Ca^2+^ levels were dose dependently decreased with 200–1600 μM limonin. However, limonin alone did not affect calcium level. Similarly, cAMP levels were decreased by up to 40% after pretreatment with 400–1600 μM limonin compared to the positive control (Figure 4B), while limonin alone did not affect cAMP level. These strongly suggest that limonin, at least, specifically inhibits eugenol-induced calcium and cAMP level in 3T3-L1 cells. However, it could not be excluded the possibility that limonin affects other signaling pathways in the cells. To determine toxicity by limonin, cell viability was assessed in cells treated with limonin. As shown in Figure 4C, up to 1.6 mM limonin did not affect cell viability. These results suggest that limonin inhibits Ca^2+^ and cAMP levels in eugenol-induced 3T3-L1 cells.

### 2.5. Limonin Decreased Eugenol-Induced Phosphorylation of PKA and CREB in 3T3-L1 Cells

In order to elucidate the effects of limonin on the OST pathway in non-neuronal cells, we examined the levels of eugenol-induced phosphorylation of PKA and CREB in limonin-pretreated cells. As shown in Figure 4D, the phosphorylation of PKA and CREB was significantly decreased by limonin in dose-dependent manner. These data suggest that limonin inhibits eugenol-induced phosphorylation of CREB and PKA in non-neuronal 3T3-L1 cells.

## 3. Experimental Section

### 3.1. Plant Material

Ethanol extract of DRC was obtained from the Korea plant extract bank at the Korea Research Institute of Bioscience and Biotechnology (KRIBB, Daejeon, Korea). The powder was dried at room temperature and then dissolved in dimethylsulfoxide (DMSO) (*v*/*v*).

### 3.2. Reagents and Antibodies

Eugenol and limonin were purchased from Sigma (St. Louis, MO, USA). The Ca^2+^ assay kit was obtained from Molecular Devices (Sunnyvale, CA, USA), and the cAMP assay kit was purchased from Enzo Life Sciences, Inc. (Farmington, NY, USA). Antibodies against phospho-protein kinase A (PKA) and phospho-cyclic AMP response element binding protein (CREB) were purchased from Cell Signaling Technology (Beverly, MA, USA), and the actin antibody was obtained from Bethyl Laboratories (Montgomery, TX, USA).

### 3.3. Cell Culture

3T3-L1 cells were purchased from American Type Culture Collection (Manassas, VA, USA) and cultured in Dulbecco’s Modified Eagle Medium (DMEM) (high glucose), which contained 10% fetal bovine serum (FBS) and 1× antibiotic-antimycotic solution (Welgene Inc., Daegu, Korea). The cells were incubated at 37 °C in the presence of 5% CO_2_.

### 3.4. Ca*^2+^* Assay

The 3T3-L1 cells were seeded in 96-well black plates and pretreated with DRC and limonin in a CO_2_ incubator for 30 min. Then, 100 μL component A buffer (Molecular Devices) was added, and the plate was covered with foil and incubated for 30 min at room temperature and then 15 min at 37 °C. Eugenol and ionomycin were automatically added in Flexstation 3 (Molecular Devices), and the Ca^2+^ level was measured according to the manufacturer’s instructions.

### 3.5. cAMP Assay

3T3-L1 cells were starved with serum-free DMEM (high glucose) media for 16–18 h, and cells were pretreated with DRC and limonin for 30 min. Cells were then treated with eugenol for 7 min and lysed with 0.1 M HCl. cAMP levels were measured using the Direct cAMP ELISA kit (Enzo Life Sciences).

### 3.6. Cell Viability Analysis

The viability of cells was determined using the Cell Counting Kit-8 Reagent (CCK-8) (Enzo Life Sciences). Cells at a concentration of 5 × 10^4^ cells/well in 200 μL culture medium were seeded in a 96-well plate. The cells were incubated for 8 h at 37 °C and 5% CO_2_ and starved for 16 h with serum-free medium, and then treated with DRC or limonin in 200 μL serum-free medium. After incubating cells with 10 μL/well CCK-8 reagent for 4 h, the absorbance of the samples was measured using a microplate reader at 450 nm.

### 3.7. Western Blot Analysis

Cells were seeded in 6-well plates and starved with serum-free DMEM for 16–18 h. The starved cells were pretreated with DRC and limonin for 30 min and treated with eugenol for 7 min. The cells were lysed with RIPA buffer (Bioseang, Seongnam-si, Korea), which contained a protease inhibitor cocktail (Roche, Basel, Switzerland) and a phosphatase inhibitor cocktail (Roche). The lysate was centrifuged at 12,000 rpm for 30 min at 4 °C. Protein concentration was measured using the SMART bicinchoninic assay (BCA) Protein Assay Kit (Intron Biotechnology, Seongnam-si, Korea). A total of 35 μg of the protein was separated on a 10% SDS-PAGE and transferred onto a nitrocellulose membrane. The membranes were blocked with 5% skim milk in 1 × Tris-buffered saline (TBS) containing Tween 20 (TBST) for 1 h at room temperature and incubated with primary antibodies at 4 °C for 16–18 h. The membranes were probed with horseradish peroxidase (HRP)-conjugated secondary antibody at room temperature for 1 h, and then immuno-reactive bands were visualized using enhanced chemiluminescence reagents (Amersham, Piscataway, NJ, USA).

### 3.8. Ultra-Performance Liquid Chromatography Tandem Mass Spectrometry (UPLC–MS/MS) Analysis

The analyses were performed using an Acquity UPLC system (Waters, Miliford, MA, USA) with Acquity UPLC BEH C18 column (2.1 mm × 100 mm, 1.7 µm). The mobile phase included 0.1% formic acid aqueous solution (Solvent A) and 0.1% formic acid in acetonitrile (Solvent B), and a gradient elution program was performed: 90% solvent A (0 min)-50% solvent A (3 min)-90% solvent A (5 min), at a flow rate of 0.3 mL/min. Column temperature was kept at 40 °C and the total run time was 5 min. The auto-sampler was conditioned at 4 °C and the injection volume was 5 μL. Identification and quantification of limonin for AP was carried out on a Waters Xevo TQ triple-quadrupole mass spectrometer equipped with electro spray ionization (ESI) mode. The ESI source was set in positive ESI mode and processed using MassLynx 4.1 (Waters) software. The quantification was performed using multiple reaction monitoring (MRM) mode of *m*/*z* 471.3 → 161.3 for limonin. The detector was operated in cone voltage 32 V, a capillary voltage 3.3 kV. The source temperature was set at 150 °C while the desolvation flow was set at 800 L/h; the desolvation gas temperature was set at 400 °C

### 3.9. Statistical Analysis

All experiments were repeated at least three times, and the data are expressed as the mean ± standard deviation (SD). Group means were compared using the non-parametric Kruskal-Wallis and Mann-Whitney analysis using SPSS (SPSS Inc., Armonk, NY, USA).

## 4. Conclusions

In summary, our study, for the first time, demonstrated that ethanol extracts of DRC and limonin inhibit the OST pathway by regulating Ca^2+^ influx, cAMP levels, and phosphorylation of CREB in non-neuronal 3T3-L1 cells. These results expand our understanding of OST pathway in non-neuronal cells and provide non-neuronal cell system to investigate novel functions of olfactory receptor(s) and its regulation by exogenous molecules such as DRC and limonin. Our study also provides an *in vitro* cell system to characterize intrinsic ligands stimulating eugenol receptor which may has physiological functions in 3T3-L1 cells.
